# The influence of the menstrual cycle on muscle injuries - a systematic review and meta-analysis

**DOI:** 10.1038/s41598-026-36763-0

**Published:** 2026-01-21

**Authors:** Yannik Guthardt, Debby Sargent, Ross Julian

**Affiliations:** 1 Department of Neuromotor Behavior and Exercise, Institute of Sport and Exercise Sciences, University of Münster, 48149 Münster, Germany; 2 Department of Biology, School of Energy, Geoscience, Infrastructure and Society, Heriot-Watt University, Edinburgh, EH14 4AS UK; 3 School of Sport and Exercise, University of Gloucestershire, Cheltenham, GL50 2RH UK

**Keywords:** Female athlete, Follicular phase, Injury occurrence, Luteal phase, Oestrogen, Team sport, Physiology, Musculoskeletal system

## Abstract

This systematic review and meta-analysis examined the relationship between menstrual cycle phases and the incidence of muscle injuries in female team sport athletes, following PRISMA 2020 and PERSiST guidelines. A comprehensive search was conducted in PubMed, Scopus, and SPORTDiscus from inception to mid-January 2024. Studies were included if they examined female team sport athletes of reproductive age with regular menstrual cycles and compared the occurrence of muscle injuries across at least two menstrual phases. Studies involving hormonal contraceptive use, medications affecting the menstrual cycle or musculoskeletal system, or menstrual dysfunction were excluded. Three studies met the inclusion criteria, involving 318 participants. Meta-analysis yielded a pooled Risk Ratio of 1.18 (95% CI: 0.75 to 1.86, *p* = 0.46) for injury risk between the luteal and follicular phases, suggesting no statistically significant association. However, the certainty of the cumulative evidence was rated as very low due to methodological limitations, including inconsistent phase classifications and reliance on imprecise methods for identifying menstrual phases. Consequently, no practical or clinical recommendations can be made at this time. Future research employing standardised, physiologically accurate methods for classifying and detecting menstrual cycle phases is necessary to better understand the potential links between hormonal fluctuations and injury risk.

**Supplementary Information:**

The online version contains supplementary material available at 10.1038/s41598-026-36763-0.

## Introduction

Over the past few decades, women’s sport has seen rapid growth in professionalism and commercialisation due to investments, structured development, and strategic planning^1–4^. This evolution has inherently led to an increase in the physical demands on athletes^5^ as well as a rise in the frequency, intensity, and competitiveness of training and competitions^6^, potentially elevating the risk of injury^7^. Given the significant health and performance-related impacts of injuries^8–12^, there is a pressing need to design and investigate effective injury-mitigation strategies.

Despite the significant underrepresentation of female participants in sports medicine research^13,14^, numerous studies have highlighted notable differences in the predominant injury types, incidence, and burden between male and female athletes^15–18^. These disparities have prompted calls for research on female-specific injury prevention programmes, including the effects of sex-specific biological factors^1,17,19^.

**Fig. 1:**
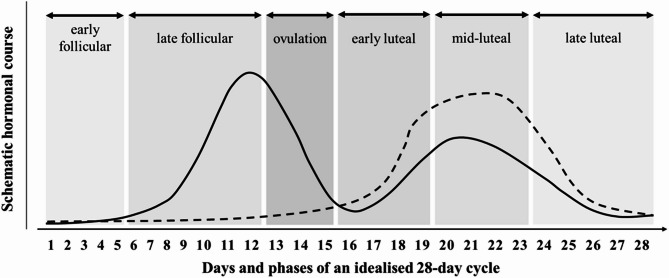
Schematic Hormonal course of an idealised 28-day cycle. solid line: oestrogen. dashed line: progesterone. Adapted from McNulty et al.^22^.

Arguably, one of the most prominent factors in eumenorrheic female athletes is the menstrual cycle, which has been shown to influence physiological functions and systems^20–22^. These cyclical changes in ovarian hormone concentrations, which can be observed in Fig. 1, lead to distinct hormonal profiles that can be used to identify and differentiate menstrual phases^23,24^. Based on an idealised 28-day cycle, the menstrual cycle can be divided into the early follicular phase (days 1–5), mid follicular phase (days 6–8), late follicular phase (days 9–13), ovulation (day 14), early luteal phase (days 15–20), mid-luteal phase (days 21–24), and the late luteal phase (days 25–28)^21^.

Systematic reviews and meta-analyses examining potential menstrual cycle-related injury risks seem to have primarily focused on the anterior cruciate ligament (ACL)^25–28^. This focus is likely due to previously demonstrated effects of ovarian hormones and the menstrual cycle on collagen formation^29,30^, the structural integrity of the ACL^31,32^, and knee laxity^31,32^. Though rare, ACL injuries often sideline athletes for several months^33,34^, and jeopardise careers^34^.

In contrast, muscle injuries are generally less severe but occur significantly more frequently. For example, in elite-level women’s football, muscle injuries in the thigh alone have been observed to occur approximately 16 times more often than ACL injuries^34^, with 15–20% of cases sidelining athletes for longer than a month^34^. Muscle strains are also the most common type of injury in both male and female athletes across various sport disciplines^34–37^. Given evidence suggesting that menstrual cycle hormones may affect soft tissue compliance^38–40^ and neuromuscular ability^41–43^, it seems plausible to assume a possible connection between the menstrual cycle and muscle injury risk. This possibility is further supported by research on changes in proprioception and pain perception during the menstrual cycle. While some studies dismiss menstrual cycle-related variations in proprioception and dynamic stability as major contributors to injury events^44^, others suggest that under high athletic demands, these factors could indeed affect injury risk^45^.

The objective of this review was to determine whether muscle injury occurrence differs across the menstrual cycle in eumenorrheic female team sport athletes. To the best of the authors’ knowledge, this represents the first systematic and comprehensive analysis of this relationship, possibly providing insights for athletes, coaches, practitioners, and researchers working to advance the understanding and prevention of muscle injuries.

## Methods

This review was pre-registered on the Open Science Framework (OSF), adheres to the PRISMA 2020 statement guidelines^46^ and covers all the items of the PRISMA checklist (https://osf.io/bys84). Further, the Prisma in Exercise, Rehabilitation, Sport medicine and SporTs science (PERSiST) guidance was followed^47^.

### Study inclusion and exclusion criteria

The PICOS framework (consisting of Population, Intervention, Comparator, Outcomes, and Study Design) guided the process of determining this review’s relevant parameters, as shown in Table 1.

**Table 1:** PICOS framework.

Parameter	Description
Population	Female team sport athletes who fulfilled the following criteria: (a) were of reproductive age (post-menarche and premenopausal), (b) had regular menstruations and ovulation cycles, (c) were non-users of any hormonal contraceptives or medications that affect the menstrual cycle or musculoskeletal system, and (d) were free from any menstrual dysfunctions (such as amenorrhea or anovulatory cycles) or other conditions that can influence their menstrual cycle and hormone profile or musculoskeletal system (such as pregnancy and relative energy deficiency syndrome). No restrictions were imposed regarding the athletes’ competition level.
Intervention	No particular intervention was investigated, but participants had to meet the population criteria above. Studies had to verify the participants’ menstrual cycle phases through established means, and the classification used for the menstrual cycle phases had to be consistent with the existing literature.
Comperator	Included studies were required to compare an outcome measure (i.e., injury number or injury rate incidence) at a minimum of two menstrual cycle phases. Injuries were defined as an occurrence that prevents an athlete from participating in training or match-play for a minimum of a day.
Outcomes	Occurrence of non-contact muscle injuries. Further, related outcomes such as injury incidence rate and muscular injuries with unclear differentiation between contact and non-contact origin were considered.
Study Design	Studies were considered for inclusion if they met the following criteria: (a) fully published in a peer-reviewed journal, (b) written in English or translated and published in English, and (c) had the primary or secondary objective of assessing the incidence of muscle injuries within the phases of the menstrual cycle. Any reviews, case reports, editorials, conference abstracts, clinical commentaries, dissertations, and unpublished studies were excluded. No restrictions were placed on the date of publication.

### Search strategy

A systematic electronic literature search was conducted independently in mid-January 2024 by two reviewers (YG and DS), each using the three databases PubMed (including MEDLINE), Scopus and SPORTDiscus to identify all relevant articles. The following search string was used: (“muscle*” OR “muscular”) AND (injur* OR strain* OR tear* OR rupture* OR “injury incidence”) AND (“menstrual cycle” OR “menstrual phase” OR “menstrual” OR “menstruation” OR “follicular phase” OR “luteal phase” OR “ovulation” OR “ovulatory” OR “sex hormone*”). Databases were searched from inception onwards. The reference lists of relevant articles obtained were hand-searched to identify further potential studies that could be added manually.

### Study Selection, data extraction and quality assessment

#### Screening and selection of eligible studies

All search results were saved and managed in the systematic review software ‘Rayyan’^48^. This tool was utilised to sort through, screen, and include qualified records. Any duplicates were automatically identified by comparing title, year, volume, and authorship. Afterwards, two reviewers (YG and DS) independently verified the accuracy of duplicates before removing them from consideration.

All the remaining articles underwent an independent two-phase screening strategy by two reviewers (YG and DS). In phase 1, the titles and abstracts were examined against the predetermined eligibility criteria. If neither the title nor the abstract of an article showed indications of meeting the inclusion criteria or met at least one of the exclusion criteria, the article was excluded in phase 1.

The full-text versions of the remaining articles were then read as phase 2 to confirm eligibility. In cases where studies were reported in multiple publications, all reports were collated. In case of disagreement on eligibility between the two reviewers, the most experienced reviewer (RJ) was consulted, and his decision was deemed final.

#### Data extraction

Two reviewers (YG and DS) independently extracted the data using a standardised template. Any discrepancies were identified and addressed through a consensus-based discussion and review of the original article. The matter was referred to the most experienced researcher (RJ) for consultation in unresolved disagreements. A comprehensive list of the extracted data items is provided on OSF (https://osf.io/bys84).

#### Quality assessment

The quality assessment was conducted independently by two reviewers (YG and DS) following the Grading of Recommendations Assessment Development and Evaluation (GRADE) system of rating quality of evidence^49^. This approach evaluates the certainty in the cumulative evidence based on five domains: risk of bias, indirectness, inconsistency, imprecision, and evidence of publication bias. Each study was individually assessed for risk of bias with the QUIPS (Quality In Prognosis Studies) appraisal tool^50^. Based on the QUIPS tool results, each study was assigned an adequate a priori quality rating of either ‘high’, ‘moderate’, ‘low’ or ‘very low’.

Following McNulty et al.^22^ and based on the recommendations of De Jonge et al.^51^, the initial rating was either maintained or downgraded based on two questions considered vital to assessing the indirectness of the research studies:

(*Q1) Was the menstrual cycle phase confirmed using blood samples or urinary ovulation detection kits?* The initial rating was maintained if the study confirmed the menstrual cycle phase using these methods. If not, the study was downgraded by one level. For example, a study initially rated as ‘high’ would be downgraded to ‘moderate’ if it did not use blood samples or urinary detection kits for confirmation.

(*Q2) Was the injury medically diagnosed by qualified experts or means?* The rating was downgraded by one level if the study did not report that qualified medical personnel recorded and diagnosed injuries. If medical staff were involved in diagnosing injuries, the Q1 rating was maintained.

Consistency was ascertained through meta-analysis, visual inspection of effect size estimates, and overlap of confidence intervals, supplemented by statistical tests for heterogeneity. Precision was judged by closely examining the number of data points supporting the relevant outcome (with outcomes based on < 5 data points being downgraded) and visual analysis of the width of the confidence intervals. The assessment of publication bias included visual examination of result patterns (funnel plot), Egger’s test and the recommendations of the GRADE workgroup^52^.

These procedures collectively led to a final certainty rating for the cumulative evidence as ‘high’, ‘moderate’, ‘low’, or ‘very low’^53^. Disagreements were resolved through discussion. If no consensus was reached, a third reviewer (RJ) made the final decision. Based on this appraisal strategy, no studies were excluded.

#### Data synthesis

To facilitate a consistent analysis across studies, each study’s menstrual cycle phases were aligned according to a predefined two-phase classification scheme commonly employed by prior menstrual cycle research^51^. The follicular phase was defined as extending from the onset of menstruation up to ovulation, and the luteal phase was considered to encompass ovulation and the remaining days until the start of the next menstrual period. The extracted data were utilised to align the phases of each eligible study to the predetermined classification for statistical analysis. This alignment was carried out based on the days of a 28-day idealised cycle length or the contextual information provided by the study in cases where the precise duration of each classified phase was ambiguous. Ovulation and its physiological characteristics were deemed to start on day 13 of a 28-day idealised menstrual cycle in line with McNulty et al.^22^ and served as a reference point for contextual alignment. Since none of the eligible studies focused exclusively on muscle injuries, the absolute number of muscle injuries was selected as the primary outcome for statistical analysis, as it was the only consistent and comparable metric across all studies.

A frequentist approach was employed for this meta-analysis, using the Risk Ratio (RR) as the primary effect size. This choice allowed for a direct comparison of the injury occurrence between the follicular and luteal phases, the latter of which acted as the ‘control condition’ across the selected studies. For each study, the RR was calculated as:

$$\:RR=\frac{Number\:of\:Injuries\:in\:Luteal\:Phase}{Number\:of\:Injuries\:in\:Follicular\:Phase}$$

The log-transformed Risk Ratio (logRR) and its associated standard error (SE) were computed to standardise the effect sizes across studies, facilitating pooling across studies. The meta-analysis was conducted using both fixed-effect (common-effect) and random-effects models to explore the impact of potential heterogeneity among studies. The fixed-effect model assumes that all studies estimate a common underlying effect size, attributing any observed differences solely to within-study variation. In contrast, the random-effects model accounts for both within and between-study heterogeneity, assuming that the true effect size may vary across studies.

In both models, weights were assigned to each study based on the inverse of the logRR’s variance. The random-effects model further adjusted the weights to account for between-study variability.

Heterogeneity across the studies was assessed using the I² statistic, which quantifies the proportion of total variation due to between-study heterogeneity. An I² value of 100% indicates maximal inconsistency.

Further, tau² was calculated using the Restricted Maximum Likelihood (REML) method to estimate the between-study variance, providing a refined measure of variability beyond what is captured by I² alone. Cochran’s Q statistic was used to test the null hypothesis that all studies evaluate the same effect. A leave-one-out sensitivity analysis was conducted to ensure the robustness of the findings.

The results were visually summarised using a forest plot, which displays each study’s individual RR and 95% confidence intervals (CIs) and the pooled effect sizes from both models. The forest plot also illustrates the weight assigned to each study, emphasising each contribution to the overall effect estimate. All analyses were conducted in the statistical software R Version 4.2.2^54^, including the use of the R package meta^55^.

## Results

### Literature search

**Fig. 2:**
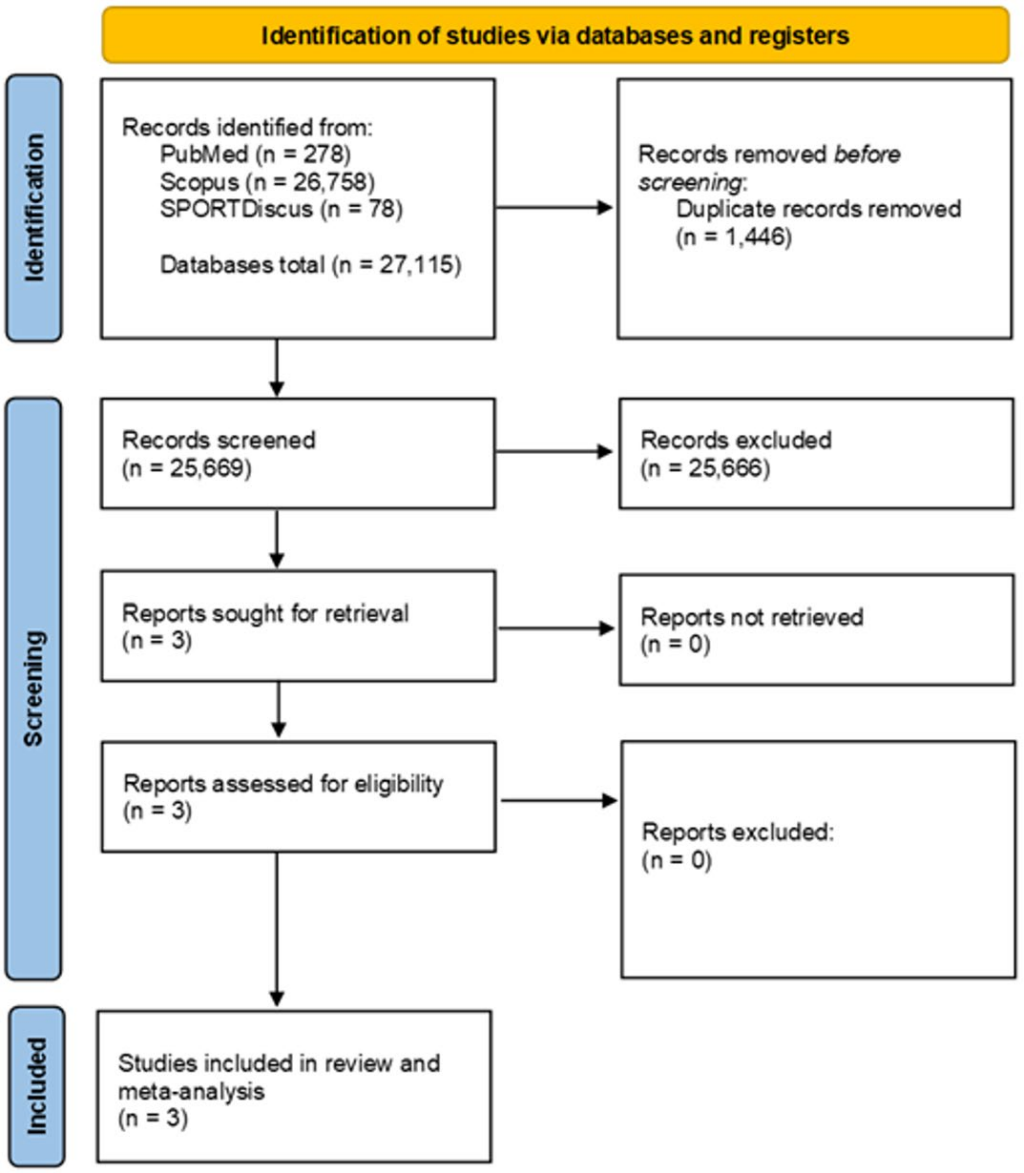
PRISMA flow diagram of the literature search.

Figure 2 illustrates the search and selection of studies in a flow chart.

### Study characteristics

The final analysis included data from three studies^56–58^. The combined total number of participants observed in two^56,58^ of the three studies was 205, while the total number of participants observed in the third study was not ascertainable. However, all relevant injury data in this study were obtained from 113 participants^57^. Two studies were conducted in professional football and one in professional futsal. All studies assessed the menstrual data via self-reporting of the players through either a mobile menstrual tracking application^58^, a combination of self-reported menstrual cycle length and a regression equation^57^, or a mixture of calendar-based counting and different mobile applications^56^. Details of the study characteristics of the included studies can be found in Table 2.

**Table 2:** Study characteristics of included Studies.

Study	Sample	Menstrual Cycle Classification	Method of MC Phase Verification	Exposure Metric	Injury Definition & Method of Diagnosis	Findings
Lago-Fuentes et al^56^.	*N* = 179 Players of the Spanish First and Second National Futsal League	*Follicular Phase* (Day 1–12) *Ovulatory Phase* (Day 13–15) *Luteal Phase* (Day 16–28)	Self-report via calendar-based counting and different apps	Total number of hours all players participated in training sessions and matches *(player-hours)*	*‘An injury that occurs during a training session or match and which causes the player to be out of the next training session or match’* No Information available	No statistical differences in the injury occurrence between the MC phases. Tendencies of higher frequencies in the *Follicular Phase*.
Martin et al^57^.	N = eight playing squads over four years, comprised of 3,947 individual player camp attendances (Injury data were obtained from *n* = 113) Players selected for the England National Football Team Under 15 s – Senior Level	*Follicular Phase* (Time between the first day of the menses and the late Follicular Phase) *Late Follicular Phase* (Day of luteinising hormone peak and the two preceding days) *Luteal Phase* (Any time point following the *Late Follicular Phase*)	Self-report via typical cycle length and regression equation	Total number of *person-days*, estimated by summing the predicted number of days in each menstrual cycle phase for the players who were injured.	*‘Occurrence which prevented a player from taking part in training or match-play for one or more days following the injury’* Recorded by each team’s medical support staff and classified using the Orchard Sports Injury Classification System by a medical professional	Muscle injuries were approximately twice as common in the *Late Follicular Phase* compared to the *Follicular Phase* and *Luteal Phase* per 1,000 person-days
Barlow et al^58^.	*N* = 26 24.1 ± 4.6 years of age Players of a professional Women’s Super League Football Club	*Phase 1* (Menstruation) *Phase 2* (Remainder of the predicted follicular phase) *Phase 3* (Majority of the luteal phase) *Phase 4* (Premenstrual window, defined as the five days before the onset of menstruation)	Self-report via a mobile tracking application that recorded menstruation days and intensity of flow	Total number of *person-days*, calculated by summing every day each individual player participated in a full training session or match	*‘An incident which prevented a player from taking part in full* *training or match-play for one or more days following the injury’* Recorded by the football club’s medical support staff and classified using the Orchard Sports Injury Classification System	Muscle injuries occurred more commonly in *Phase 3* and *Phase 4* than in *Phase 1* and *2.* Compared to *Phase 1*, muscle injuries occurred three times more likely in *Phase 2*, five times more likely in *Phase 3*, and over six times more likely in *Phase 4* per 1,000 person-days

### Study findings

Muscle injury occurrence across the studies showed variation between menstrual cycle phases, with inconsistent results reported. One study^57^ observed a considerable increase in muscular injuries during the late follicular phase, as defined in their research. In contrast, another study reported the highest injury rates during the luteal phase, particularly in the premenstrual window^58^. A third study^56^ indicated a tendency toward higher injury frequencies in the follicular phase compared to the other phases they defined, although these differences were not statistically significant. Detailed findings from each study are presented in Table 2.

### Quality assessment of included studies

Three studies (100%) were considered to be at high risk of bias after the risk of bias assessment of the individual study level obtained from the QUIPS tool and additional questions regarding the menstrual cycle phase and injury detection and verification. All studies were allocated an a priori rating of ‘moderate’ but downgraded based on the additional questions (Q1) and (Q2) regarding the method of menstrual phase verification and injury diagnosis and recording. Details of the domain-based risk of bias assessment from the QUIPS tool and (Q1) and (Q2) can be found on OSF (https://osf.io/bys84).

No asymmetry was observed following a visual examination of the funnel plot. Egger’s test also indicated no detectable publication bias (*p* = 0.92). However, because all included papers are observational studies, the recommendations of the GRADE workgroup were followed, and publication bias was considered to be inherently substantial^52^. Based on the GRADE approach, the certainty of the cumulative evidence of this review was assessed to be ‘very low’^53^.

### Meta-Analysis results

**Fig. 3:**
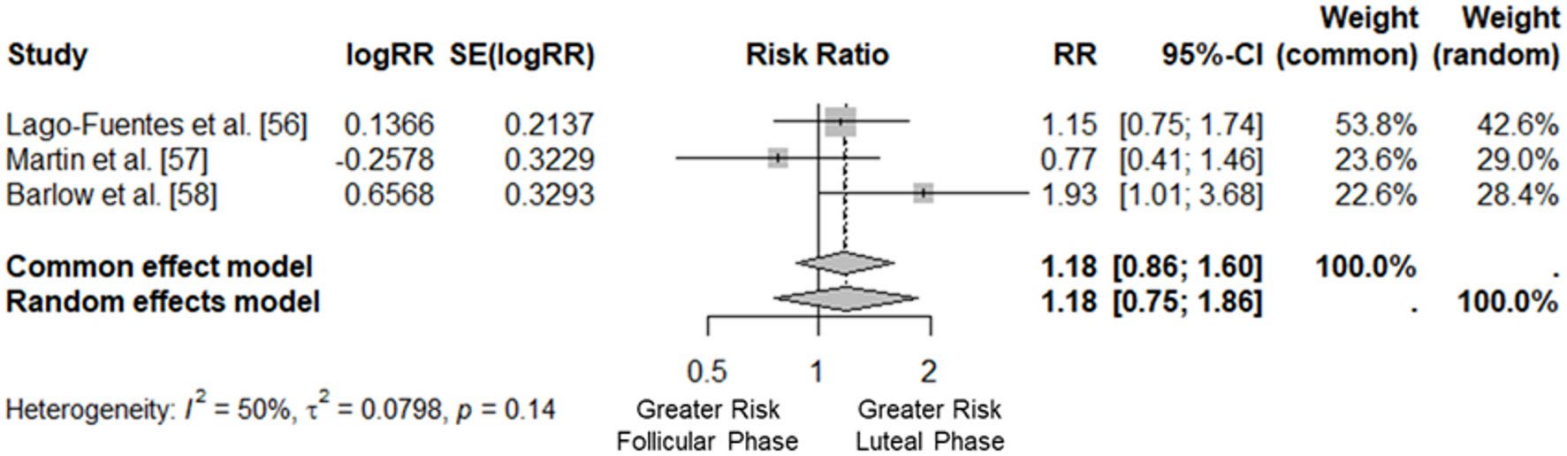
Forest plot of the meta-analysis results.

The common-effect model meta-analysis revealed a pooled Risk Ratio of 1.18 (95% CI: 0.86 to 1.60, z = 1.03, *p* = 0.30), suggesting no statistically significant difference in injury occurrence between the luteal and follicular phases. The random-effects model, which accounts for between-study heterogeneity, produced a similar pooled RR of 1.18 (95% CI: 0.75 to 1.86, z = 0.73, *p* = 0.46), further indicating the absence of a significant association between the menstrual cycle phase and muscle injury occurrence. The results of the meta-analysis are visualised in Fig. 3.

The heterogeneity across studies was moderate, with an I² of 49.5% (95% CI: 0.0% to 85.3%) and a tau² of 0.08 (95% CI: 0.00 to 7.21). Cochran’s Q test for heterogeneity yielded a Q value of 3.96 (d.f. = 2, *p* = 0.14).

The leave-one-out sensitivity analysis to examine each study’s influence on the overall results elicited varying RRs from 1.01 to 1.40, with corresponding 95% CIs that consistently overlapped and included the null value (RR = 1.0), indicating no significant effect. The pooled RR increased slightly to 1.22 (95% CI: 0.50 to 2.98, z = 0.43, *p* = 0.67), and the highest heterogeneity was observed (I² = 74.6%, Q = 3.93, d.f. = 1, *p* = 0.05) with the exclusion of the Lago-Fuentes et al. paper^56^, suggesting a significant impact on the overall results. In contrast, the lowest heterogeneity was observed with an I² of 3.6% (Q = 1.04, d.f. = 1, *p* = 0.31) with the exclusion of the Barlow et al. study^58^, resulting in a pooled RR of 1.01 (95% CI: 0.71 to 1.45, z = 0.07, *p* = 0.94).

## Discussion

This systematic review and meta-analysis sought to elucidate whether the menstrual cycle and its phases have a significant impact on the occurrence of muscle injuries in eumenorrheic female team sport athletes. The premise that there might be variability in muscle injuries across the menstrual cycle is grounded in previous findings on the effects of the inherent hormonal fluctuations on musculoskeletal function introduced at the beginning of this review, including altered soft tissue plasticity^38–40^, impacted collagen metabolism^29,30^, and potentially distorted neuromuscular control^41–43^ and proprioception^45^.

Despite the biological and theoretical plausibility, the current meta-analysis did not find a statistically significant difference in the distribution of muscle injuries between the follicular and luteal phases of the menstrual cycle. The pooled Risk Ratio of 1.18, derived from both fixed-effect and random-effects models, indicates a non-significant association between the menstrual cycle phase and the instance of muscular injuries. This suggests that while hormonal fluctuations may influence musculoskeletal properties, these changes may not translate into a measurable difference in injury risk across the menstrual cycle. Notably, this conflicts with the athletes’ perception, as over half of female athletes report a changed perceived risk of injury throughout the menstrual cycle^59^. When examining the RRs and conclusions from the individual studies, discordant and conflicting results become apparent, mirroring previous research predominantly centred on ACL injuries with similarly inconsistent findings^27,28^. While these findings provide valuable information and appear to loosely align with existing evidence from similar research, the limited available literature reduces statistical power and warrants caution when interpreting the pooled estimate and overall results. Accordingly, the heterogeneity tests should be regarded as uncertain, and the non-significant Cochrane Q should not be taken as evidence of homogeneity due to the small number of included studies^60^. Furthermore, the meta-analysis was based on aggregated study-level data, whereas the original studies employed repeated-measures designs in which individual athletes contributed multiple observations across menstrual phases. The assumption of independence in the analytical model may therefore have introduced bias by failing to account for within-subject correlation.

### Limitations

Beyond these statistical concerns, several overarching limitations in the review and the included studies further complicate interpretation. The three paramount limitations are (a) the inconsistent and dissimilar classification of the menstrual cycle phases across scientific literature, (b) the resulting need to map and align to a standardised system for statistical analysis, which may obscured or diluted true effects and nuances, and (c) the methods used by the included studies to detect and verify the menstrual cycle phases.

**Fig. 4:**
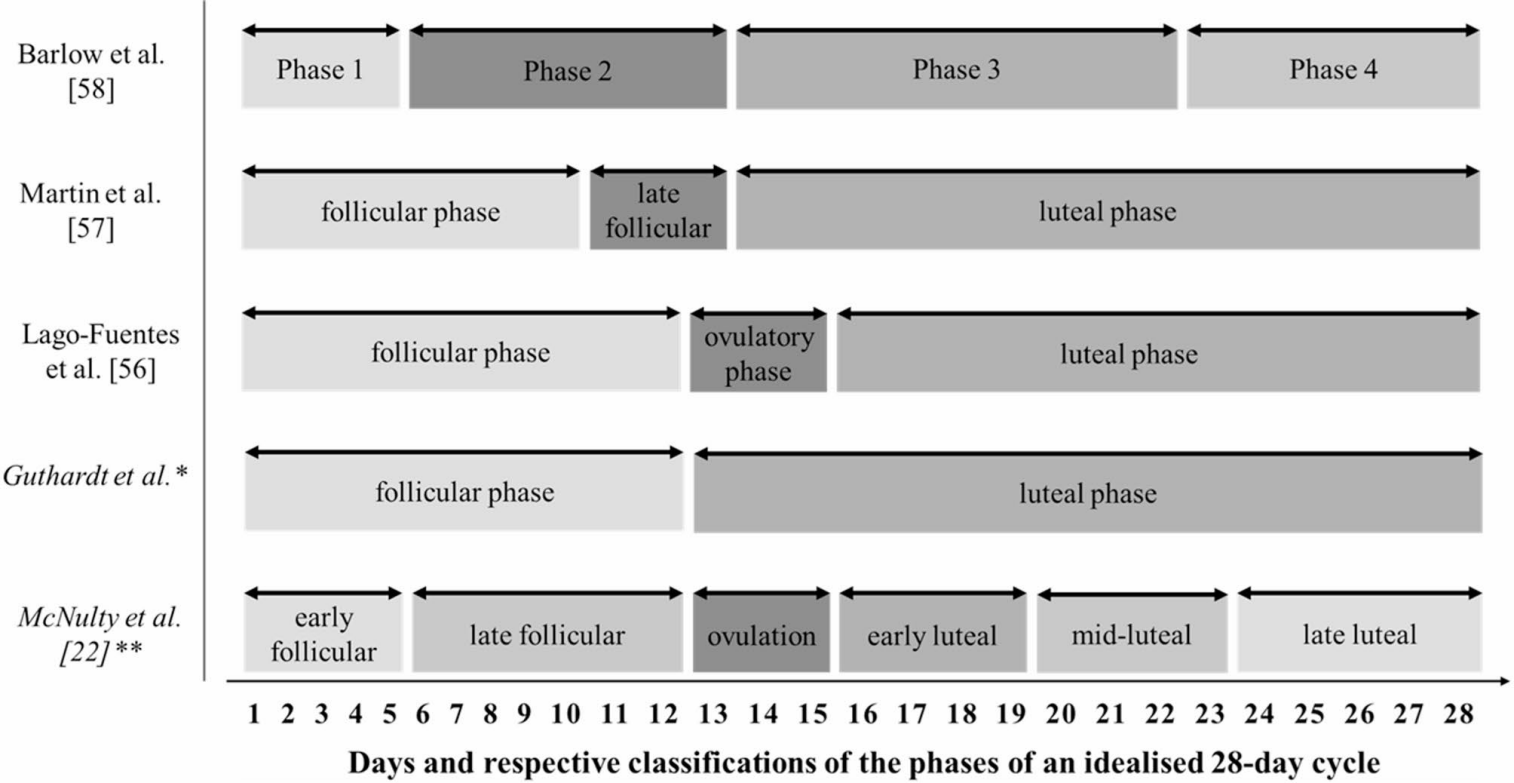
Variety in Menstrual Cycle Classifications Across Included Studies and Scientific Literature. The first three studies were included in the meta-analysis. *Classification scheme for the statistical analysis of this review. **Classification scheme of McNulty et al.^22^.

The inconsistent classification of menstrual phases across studies poses significant challenges for comparing and interpreting findings. There is considerable variation in how the menstrual cycle is divided, with differences in the number of phases, terminology, duration, and specific time points assigned to each phase. This variety is visualised in Fig. 4. As a result of this, events occurring at the same specific time point in the menstrual cycle might be interpreted and reported in completely unrelated and conflicting phases across the scientific literature. This issue is exacerbated when attempting to synthesise evidence and data across studies, as the heterogeneity introduced by different phase definitions complicates meta-analytical approaches, increasing the risk of biased, erroneous, or misleading findings.

In order to statistically synthesise and analyse the data of the included studies for this review, all respective phase classifications were aligned to a two-phase system, creating a follicular phase and a luteal phase. While this allowed for statistical analysis across the findings of the included studies and aligns with the majority of previous menstrual cycle research, which typically distinguishes and compares these two phases^51^, it is vital to note that it does not accurately reflect the complex dynamic of the typical menstrual cycle, involving multiple fluctuations in oestrogen and progesterone levels at various stages.

Given the non-linear relationship between circulating hormones and their physiological effects, these distinct changes in the hormonal milieu and specific hormonal peaks may have substantial impacts on key biological factors related to injury risk, including muscle proteostasis and tissue stiffness^61^. This may be further magnified by evidence that mRNA and protein levels of oestrogen and progesterone receptors vary across the course of the menstrual cycle^62^. Thus, reducing the menstrual cycle to unsuitably broad and generalised phases, as was possibly done for the analysis, may overlook critical hormonal fluctuations and their potential effects.

Furthermore, the identification and verification of the menstrual cycle phases in the included studies were substantially based on self-reported data and calendar-based counting methods, which are inherently prone to inaccuracies. These methods, while convenient and considerably more cost-effective compared to hormonal assays, rely on temporal estimates and are susceptible to miscalculations and fallacious reflections of an individual’s menstrual cycle due to a wide range of factors, including inter- and intra-individual variability in the cycle length and ovulation timing, as well as further unrecognised variability in the cycle induced through stress, exercise, illness, and changes in body weight^63–65^. Additionally, these methods typically define menstrual cycle phases based on self-reported data regarding the onset of menstruation. However, it is essential to recognise that the occurrence of regular menstrual bleeding does not necessarily indicate an ovulatory cycle with the corresponding typical hormonal course^51,66^. As a result, the sole use of calendar-based methods for phase identification is considered insufficiently accurate for reliably determining the menstrual cycle phases^51^.

Several confounding factors may have obscured potential associations between menstrual cycle phases and muscle injuries. These possible confounders include, but are not limited to, variations in training load, cumulative training and game exposure, psychophysiological influences such as stress and fatigue, and, most notably, the athletes’ previous injury history, given that previous muscle injury is widely recognised as the most significant risk factor for future muscle injury^67^. This complexity is exemplified by Lago-Fuentes et al.^56^, who observed a higher incidence of injuries during the season’s first quarter, attributed this to increased training load during that period. Further, they reported that over 60% of injuries occurred in the later stages of training sessions, highlighting the possible role of fatigue. The use of inconsistent exposure metrics in the included studies, ranging from actual person-days^58^ to estimated phase durations^57^ and player-hours without phase breakdowns^56^, hindered normalising for exposure time and the use of a preferred incidence rate ratio, further limiting the interpretability and generalisability of this review’s results.

Moreover, the Lago-Fuentes et al. study^56^ collected data over two full seasons, but only excluded and controlled for hormonal contraceptives in the second season. The relatively low percentage (5%) of excluded hormonal contraceptive users in the second season might suggest a limited impact on the data from the first season, which ultimately led to the study’s inclusion after consensus-based discussion. However, it is important to acknowledge that a small, albeit unknown, proportion of data from participants using hormonal contraceptives was included in this review’s final analysis.

Similarly, the authors cannot guarantee that all injury data were derived from non-contact events. While muscle injuries, particularly strains and tears, are typically attributed to intrinsic factors, the injury mechanism, stemming from excessive tensile or shear forces leading to the failure of muscle fibres and their surrounding connective tissue, can also result from external forces such as traumatic contact^67^. Any muscle injuries caused by external contact included in this review might have skewed the results.

Furthermore, all muscular injuries were pooled together without distinction regarding their specific mechanism (e.g., strain versus tear) or severity (e.g., a mild Grade I strain versus a complete muscle rupture). The lack of this granularity may obscure physiologically relevant effects that may be limited to certain injury types or severities, such as possible hormonal influences on tissue compliance that affect strain injuries more than ruptures. This omission further constrains interpretability and comparability across studies and may mask clinically meaningful associations. Additionally, variation in injury classification and verification methods among the included studies exacerbates this issue and echoes broader inconsistencies highlighted in a recent scoping review^68^, which emphasised that heterogeneity in injury definitions and measurement across research on menstrual cycle phases and injuries further complicates comparative research efforts in this field.

### Strengths

Despite these limitations, this review has notable strengths. Its transparent approach, highlighted by pre-registration on the Open Science Framework, strict adherence to PRISMA 2020 and PERSiST guidelines, and the use of the GRADE system to assess evidence certainty, sets a high standard of scientific rigour. These measures improve reproducibility and reliability, ensuring that the findings, although limited, offer a strong foundation for guiding future research.

### Recommendations for future research

The findings and limitations of this review highlight the need for more robust and well-designed research to explore the relationship between the menstrual cycle and muscle injury risk. Future research should prioritise the use of hormonal assays, such as serum measurements of oestrogen and progesterone, as well as urinary ovulation detection kits, to accurately verify menstrual cycle phases. Adopting the methodological recommendations, guidelines, and protocols established by DeJonge et al.^51^ to strengthen quality across the scientific body. In addition, the classification scheme of the menstrual cycle needs to be standardised to simplify evidence synthesis and mitigate the loss of intricate data details through necessary phase alignment. The authors recommend using the classification system previously used by McNulty et al.^22^ (visualised in Fig. 4), given that it includes the three most distinctive hormonal profiles of the menstrual cycle, namely, the early follicular phase with low oestrogen and low progesterone, the late follicular phase with high oestrogen and low progesterone, and the mid-luteal phase with high oestrogen and high progesterone, as reported by DeJonge and colleagues^51^.

This approach may uncover more subtle associations between hormonal fluctuations and injury risk that were not detectable in the current analysis. It could also clarify the extent to which hormonal effects, such as progesterone’s commonly associated stabilising influence on collagen^69,70^ and oestrogen’s regulatory role in tissue metabolism^61,71^, contribute to injury susceptibility in this context. By elucidating these relationships, this approach could serve as a foundation for investigating potential causative links, rather than mere associations, between the hormonal fluctuations of the menstrual cycle and muscular injuries.

The authors acknowledge that these recommendations for future research pose substantial challenges, including the invasiveness of procedures, associated costs, and the need for specialised facilities and personnel, which may seem discouraging. However, research in this area is critically needed, and finding a compromise is essential for advancing the field. Based on DeJonge and colleagues’ recommendations^51^, this review suggests that studies unable to incorporate direct hormone measurements may still achieve sufficient accuracy by employing large sample sizes and using urinary detection kits to measure luteinising hormone.

It is important to recognise that categorising injury events into specific menstrual cycle phases will inevitably involve some degree of approximation, estimation, and retrospective day-counting. However, the absence of direct physiological measurements significantly amplifies the risk of inaccuracies, misrepresentations, and misclassifications, thereby undermining the ability to draw robust conclusions about the influence of menstrual cycle phases on injury occurrence. Ultimately, the accuracy of scientific findings must be solid and reliable, ensuring that their applicability to real-world settings is not compromised by a foundation built on uncertainty.

## Conclusion

This meta-analysis did not reveal a statistically significant association between menstrual cycle phases and muscle injury incidence in female team sport athletes. However, this finding should be interpreted as a reflection of the limitations in the available evidence rather than a definitive absence of a relationship. The divergence between this finding and athletes’ reports of perceived cyclical vulnerability, along with the limitations of the included studies, suggests that the true relationship may be more complex than currently understood. Although the theoretical basis for hormonal influences on tissue properties is compelling, methodological limitations, most notably inconsistent phase classification and the use of imprecise, self-reported measures for menstrual phase detection, may have masked subtle but clinically relevant effects. Future research in this field is needed and should be obliged to adopt standardised, physiologically accurate methods for detecting and classifying menstrual phases to enhance the comparability of studies and ultimately lead to more effective injury prevention strategies tailored to female athletes.

## Supplementary Information

Below is the link to the electronic supplementary material.

Supplementary Material 1

Supplementary Material 2

## Data Availability

All data generated or analysed during this study are available in the Open Science Framework repository (https://osf.io/bys84).

## References

[1] Fédération Internationale de Football Association. Women’s Football Strategy. FIFA Strasse 20, P.O Box 8044 Zurich, Switzerland (2018).

[2] International Olympic Committee. Factsheet: Women in the Olympic Movement 2023.

[3] The Football Association. Inspiring Positive Change - The FA Strategy for Women’s and Girl’s Football: 2020–2024 2020.

[4] Women’s Sport Trust. Visibility Uncovered 2023 - The Year In Review 2024.

[5] Martínez-Lagunas, V., Niessen, M. & Hartmann, U. Women’s football: player characteristics and demands of the game. *J. Sport Health Sci.* **3** (4), 258–272 (2014). https://www.sciencedirect.com/science/article/pii/S2095254614000982

[6] Klein, M-L. Women’s Football Leagues in Europe: Organizational and Economic Perspectives. In: Pfister G, Pope S, eds. Female Football Players and Fans. London: Palgrave Macmillan UK 2018:77–101.

[7] Orejel Bustos, A. et al. Overuse-Related injuries of the musculoskeletal system: systematic review and quantitative synthesis of injuries, Locations, risk factors and assessment techniques. *Sens. (Basel)*. **21** (7). 10.3390/s21072438 (2021). [published Online First: 1 April 2021]. 10.3390/s21072438 PMC8037357 33916269

[8] Lohmander, L. S. et al. High prevalence of knee osteoarthritis, pain, and functional limitations in female soccer players twelve years after anterior cruciate ligament injury. *Arthritis Rheum.* **50** (10), 3145–3152 (2004). 15476248 10.1002/art.20589

[9] Drew, M. K., Raysmith, B. P. & Charlton, P. C. Injuries impair the chance of successful performance by sportspeople: a systematic review. *Br. J. Sports Med.* **51** (16), 1209–1214. 10.1136/bjsports-2016-096731 (2017). [published Online First: 26 April 2017]. 28446456 10.1136/bjsports-2016-096731

[10] Haugen, E. Athlete mental health & psychological impact of sport injury. *Operative Techniques Sports Med.* **30** (1), 1–10 10.1016/j.otsm.2022.150898 (2022).

[11] Eliakim, E. et al. Estimation of injury costs: financial damage of english premier league teams’ underachievement due to injuries. *BMJ Open. Sport Exerc. Med.* **6** (1), e000675. 10.1136/bmjsem-2019-000675 (2020). [published Online First: 20 May 2020]. 32537241 10.1136/bmjsem-2019-000675 PMC7247414

[12] Pulici, L. et al. Injury burden in professional European football (Soccer): systematic Review, Meta-Analysis, and economic considerations. *Clin. J. Sport Med.*10.1097/JSM.0000000000001107 (2022). [published Online First: 22 November 2022]. 36730365 10.1097/JSM.0000000000001107

[13] Emmonds, S., Heyward, O. & Jones, B. The challenge of applying and undertaking research in female sport. *Sports Med. Open.* **5** (1), 51. 10.1186/s40798-019-0224-x (2019). [published Online First: 12 December 2019]. 31832880 10.1186/s40798-019-0224-x PMC6908527

[14] Costello, J. T., Bieuzen, F. & Bleakley, C. M. Where are all the female participants in sports and exercise medicine research? *Eur. J. Sport Sci.* **14** (8), 847–851 (2014). [published Online First: 25 April 2014]. 24766579 10.1080/17461391.2014.911354

[15] Hollander, K. et al. Sex-Specific differences in running injuries: A systematic review with Meta-Analysis and Meta-Regression. *Sports Med.* **51** (5), 1011–1039. 10.1007/s40279-020-01412-7 (2021). [published Online First: 12 January 2021]. 33433864 10.1007/s40279-020-01412-7 PMC8053184

[16] Klein, C., Henke, T. & Platen, P. Injuries in football (soccer)—a systematic review of epidemiology and aetiological aspects. *Ger. J. Exerc. Sport Res.* **48** (3), 309–322. 10.1007/s12662-018-0530-3.pdf (2018). https://link.springer.com/content/pdf/

[17] Larruskain, J. et al. A comparison of injuries in elite male and female football players: A five-season prospective study. *Scand. J. Med. Sci. Sports*. **28** (1), 237–245. 10.1111/sms.12860 (2018). [published Online First: 27 March 2017]. 28207979 10.1111/sms.12860

[18] Lin, C. Y. et al. Sex differences in common sports injuries. *PM R*. **10** (10), 1073–1082. 10.1016/j.pmrj.2018.03.008 (2018). [published Online First: 14 March 2018]. 29550413 10.1016/j.pmrj.2018.03.008 PMC6138566

[19] Edouard, P. et al. Sex differences in injury during top-level international athletics championships: surveillance data from 14 championships between 2007 and 2014. *Br. J. Sports Med.* **49** (7), 472–477. 10.1136/bjsports-2014-094316 (2015). [published Online First: 24 January 2015]. 25618889 10.1136/bjsports-2014-094316

[20] Driver, H. S. et al. The menstrual cycle effects on sleep. *Sleep Med. Clin.* **3** (1), 1–11 (2008).

[21] Pitchers, G. & Elliott-Sale, K. Considerations for coaches training female athletes. *Prof. Strength. Cond*. **55**, 19–30 (2019).

[22] McNulty, K. L. et al. The effects of menstrual cycle phase on exercise performance in eumenorrheic women: A systematic review and Meta-Analysis. *Sports Med.* **50** (10), 1813–1827 (2020). 32661839 10.1007/s40279-020-01319-3 PMC7497427

[23] Janse de Jonge, X. A. K. Effects of the menstrual cycle on exercise performance. *Sports Med.* **33** (11), 833–851 (2003). 12959622 10.2165/00007256-200333110-00004

[24] Mihm, M., Gangooly, S. & Muttukrishna, S. The normal menstrual cycle in women. *Anim. Reprod. Sci.* **124** (3–4), 229–236. 10.1016/j.anireprosci.2010.08.030 (2011). [published Online First: 3 September 2010]. 20869180 10.1016/j.anireprosci.2010.08.030

[25] Hewett, T. E., Zazulak, B. T. & Myer, G. D. Effects of the menstrual cycle on anterior cruciate ligament injury risk: a systematic review. *Am. J. Sports Med.* **35** (4), 659–668. 10.1177/0363546506295699 (2007). [published Online First: 9 February 2007]. 17293469 10.1177/0363546506295699

[26] Moriceau, J. et al. The influence of the menstrual cycle and oral contraceptives on knee laxity or anterior cruciate ligament injury risk: A systematic review. *Appl. Sci.* **12** (24), 12627 (2022).

[27] Somerson, J. S. et al. The menstrual cycle May affect anterior knee laxity and the rate of anterior cruciate ligament rupture: A systematic review and Meta-Analysis. *JBJS Rev.* **7** (9), e2 (2019). 31490339 10.2106/JBJS.RVW.18.00198

[28] Dos’Santos, T. et al. Effects of the menstrual cycle phase on anterior cruciate ligament neuromuscular and Biomechanical injury risk surrogates in eumenorrheic and naturally menstruating women: A systematic review. *PLoS One*. **18** (1), e0280800. 10.1371/journal.pone.0280800 (2023). [published Online First: 26 January 2023]. 36701354 10.1371/journal.pone.0280800 PMC9879429

[29] Yu, W. D. et al. Combined effects of Estrogen and progesterone on the anterior cruciate ligament. *Clin. Orthop. Relat. Res.* **383**, 268–281. 10.1097/00003086-200102000-00031 (2001). 10.1097/00003086-200102000-00031 11210964

[30] Yu, W. D. et al. Effect of Estrogen on cellular metabolism of the human anterior cruciate ligament. *Clin. Orthop. Relat. Res.* **366**, 229–238. 10.1097/00003086-199909000-00030 (1999). 10.1097/00003086-199909000-00030 10627740

[31] Park, S-K. et al. Changing hormone levels during the menstrual cycle affect knee laxity and stiffness in healthy female subjects. *Am. J. Sports Med.* **37** (3), 588–598. 10.1177/0363546508326713 (2009). [published Online First: 27 January 2009]. 19174550 10.1177/0363546508326713

[32] Park, S-K. et al. Relationship between knee joint laxity and knee joint mechanics during the menstrual cycle. *Br. J. Sports Med.* **43** (3), 174–179. 10.1136/bjsm.2008.049270 (2009). [published Online First: 26 August 2008]. 18728055 10.1136/bjsm.2008.049270

[33] Ekstrand, J. et al. Time before return to play for the most common injuries in professional football: a 16-year follow-up of the UEFA elite club injury study. *Br. J. Sports Med.* **54** (7), 421–426. 10.1136/bjsports-2019-100666 (2020). [published Online First: 10 June 2019]. 31182429 10.1136/bjsports-2019-100666 PMC7146935

[34] Hallén, A. et al. UEFA women’s elite club injury study: a prospective study on 1527 injuries over four consecutive seasons 2018/2019 to 2021/2022 reveals thigh muscle injuries to be most common and ACL injuries most burdensome. *Br. J. Sports Med.* **58** (3), 128–135. 10.1136/bjsports-2023-107133 (2024). [published Online First: 7 February 2024]. 38182274 10.1136/bjsports-2023-107133 PMC10894819

[35] Ekstrand, J., Hägglund, M. & Waldén, M. Injury incidence and injury patterns in professional football: the UEFA injury study. *Br. J. Sports Med.* **45** (7), 553–558. 10.1136/bjsm.2009.060582 (2011). [published Online First: 23 June 2009]. 19553225 10.1136/bjsm.2009.060582

[36] Jacobsson, J. et al. Injury patterns in Swedish elite athletics: annual incidence, injury types and risk factors. *Br. J. Sports Med.* **47** (15), 941–952. 10.1136/bjsports-2012-091651 (2013). [published Online First: 29 March 2013]. 23543425 10.1136/bjsports-2012-091651

[37] Panagodage Perera, N. K. et al. The incidence, prevalence, nature, severity and mechanisms of injury in elite female cricketers: A prospective cohort study. *J. Sci. Med. Sport*. **22** (9), 1014–1020. 10.1016/j.jsams.2019.05.013 (2019). [published Online First: 25 May 2019]. 31182262 10.1016/j.jsams.2019.05.013

[38] Ham, S. et al. Greater muscle stiffness during contraction at menstruation as measured by Shear-Wave elastography. *Tohoku J. Exp. Med.* **250** (4), 207–213 (2020). 32238619 10.1620/tjem.250.207

[39] Sung, E-S. & Kim, J-H. The difference effect of Estrogen on muscle tone of medial and lateral thigh muscle during ovulation. *J. Exerc. Rehabil*. **14** (3), 419–423. 10.12965/jer.1836110.055 (2018). [published Online First: 30 June 2018]. 30018928 10.12965/jer.1836110.055 PMC6028216

[40] Yim, J., Petrofsky, J. & Lee, H. Correlation between mechanical properties of the ankle muscles and postural sway during the menstrual cycle. *Tohoku J. Exp. Med.* **244** (3), 201–207 (2018). 29540626 10.1620/tjem.244.201

[41] Weidauer, L. et al. Neuromuscular performance changes throughout the menstrual cycle in physically active females. *J. Musculoskelet. Neuronal Interact.* **20** (3), 314–324 (2020). 32877968 PMC7493438

[42] Graja, A. et al. Physical, Biochemical, and neuromuscular responses to repeated sprint exercise in eumenorrheic female handball players: effect of menstrual cycle phases. *J. Strength. Cond Res.* **36** (8), 2268–2276. 10.1519/JSC.0000000000003556 (2022). [published Online First: 10 March 2020]. 32168179 10.1519/JSC.0000000000003556

[43] Ansdell, P. et al. Menstrual cycle-associated modulations in neuromuscular function and fatigability of the knee extensors in eumenorrheic women. *J. Appl. Physiol. (1985)*. **126** (6), 1701–1712. 10.1152/japplphysiol.01041.2018 (2019). [published Online First: 7 March 2019]. 30844334 10.1152/japplphysiol.01041.2018

[44] Legerlotz, K. et al. Constant performance in balance and proprioception tests across the menstrual cycle - a pilot study in well trained female ice hockey players on hormonal contraception. *Health Sci. Rep.* **1** (1), e18. 10.1002/hsr2.18 (2018). [published Online First: 24 November 2017]. 30623036 10.1002/hsr2.18 PMC6266417

[45] Frizziero, A. et al. Changes in proprioceptive control in the menstrual cycle: a risk factor for injuries? A Proof-of-Concept study. *Muscle Ligaments Tendons J.* **13** (03), 360 (2023).

[46] Page, M. J. et al. The PRISMA 2020 statement: an updated guideline for reporting systematic reviews. *BMJ* **372**, n71. 10.1136/bmj.n71 (2021). [published Online First: 29 March 2021]. 33782057 10.1136/bmj.n71 PMC8005924

[47] Ardern, C. L. et al. Implementing the 27 PRISMA 2020 statement items for systematic reviews in the sport and exercise medicine, musculoskeletal rehabilitation and sports science fields: the persist (implementing Prisma in Exercise, Rehabilitation, sport medicine and sports science) guidance. *Br. J. Sports Med.* **56** (4), 175–195. 10.1136/bjsports-2021-103987 (2022). [published Online First: 8 October 2021]. 34625401 10.1136/bjsports-2021-103987 PMC8862073

[48] Ouzzani, M. et al. Rayyan-a web and mobile app for systematic reviews. *Syst. Rev.* **5** (1), 210. 10.1186/s13643-016-0384-4 (2016). [published Online First: 5 December 2016]. 27919275 10.1186/s13643-016-0384-4 PMC5139140

[49] Guyatt, G. et al. GRADE guidelines: 1. Introduction-GRADE evidence profiles and summary of findings tables. *J. Clin. Epidemiol.* **64** (4), 383–394. 10.1016/j.jclinepi.2010.04.026 (2011). [published Online First: 31 December 2010]. 21195583 10.1016/j.jclinepi.2010.04.026

[50] Hayden, J. A. et al. Assessing bias in studies of prognostic factors. *Ann. Intern. Med.* **158** (4), 280–286 (2013). 23420236 10.7326/0003-4819-158-4-201302190-00009

[51] Janse, D. E., Jonge, X., Thompson, B. & Han, A. Methodological recommendations for menstrual cycle research in sports and exercise. *Med. Sci. Sports Exerc.* **51** (12), 2610–2617 (2019). 31246715 10.1249/MSS.0000000000002073

[52] Guyatt, G. H. et al. GRADE guidelines: 5. Rating the quality of evidence–publication bias. *J. Clin. Epidemiol.* **64** (12), 1277–1282. 10.1016/j.jclinepi.2011.01.011 (2011). [published Online First: 30 July 2011]. 21802904 10.1016/j.jclinepi.2011.01.011

[53] Balshem, H. et al. GRADE guidelines: 3. Rating the quality of evidence. *J. Clin. Epidemiol.* **64** (4), 401–406. 10.1016/j.jclinepi.2010.07.015 (2011). [published Online First: 5 January 2011]. 21208779 10.1016/j.jclinepi.2010.07.015

[54] R Core Team. *R: A Language and Environment for Statistical* (R Foundation for Statistical Computing, 2025).

[55] Balduzzi, S., Rücker, G. & Schwarzer, G. How to perform a meta-analysis with R: a practical tutorial. *Evid. Based Ment Health*. **22** (4), 153–160. 10.1136/ebmental-2019-300117 (2019). [published Online First: 28 September 2019]. 31563865 10.1136/ebmental-2019-300117 PMC10231495

[56] Lago-Fuentes, C. et al. Follicular phase of menstrual cycle is related to higher tendency to suffer from severe injuries among elite female futsal players. *Phys. Ther. Sport*. **52**, 90–96 (2021). [published Online First: 21 August 2021]. 34450561 10.1016/j.ptsp.2021.08.008

[57] Martin, D. et al. Injury incidence across the menstrual cycle in international footballers. *Front. Sports Act. Living*. **3**, 616999. 10.3389/fspor.2021.616999 (2021). [published Online First: 1 March 2021]. 33733235 10.3389/fspor.2021.616999 PMC7956981

[58] Barlow, A. et al. Injury Incidence, severity and type across the menstrual cycle in female footballers: A prospective three season cohort study. *Med. Sci. Sports Exerc.*10.1249/MSS.0000000000003391 (2024). [published Online First: 8 February 2024]. 38227488 10.1249/MSS.0000000000003391

[59] Hayward, E. et al. Role of the menstrual cycle on performance and injury risk: A survey of female professional rugby players in the united Kingdom. *Int. J. Environ. Res. Public. Health*. **21** (2). 10.3390/ijerph21020150 (2024). [published Online First: 29 January 2024]. 10.3390/ijerph21020150 PMC10888092 38397641

[60] Higgins, J. P. T. (ed) *Cochrane Handbook for Systematic Reviews of Interventions* (Wiley-Blackwell, 2019).

[61] Chidi-Ogbolu, N. & Baar, K. Effect of Estrogen on musculoskeletal performance and injury risk. *Front. Physiol.* **9**, 1834. 10.3389/fphys.2018.01834 (2018). [published Online First: 15 January 2019]. 30697162 10.3389/fphys.2018.01834 PMC6341375

[62] Ekenros, L. et al. Expression of sex steroid hormone receptors in human skeletal muscle during the menstrual cycle. *Acta Physiol. (Oxf)*. **219** (2), 486–493. 10.1111/apha.12757 (2017). [published Online First: 9 August 2016]. 27438889 10.1111/apha.12757

[63] Barrett, E. S. et al. A factor analysis approach to examining relationships among ovarian steroid concentrations, gonadotrophin concentrations and menstrual cycle length characteristics in healthy, cycling women. *Hum. Reprod.* **28** (3), 801–811. 10.1093/humrep/des429 (2013). [published Online First: 18 December 2012]. 23250924 10.1093/humrep/des429 PMC3571500

[64] Harlow, S. D. & Matanoski, G. M. The association between weight, physical activity, and stress and variation in the length of the menstrual cycle. *Am. J. Epidemiol.* **133** (1), 38–49 (1991). 1983897 10.1093/oxfordjournals.aje.a115800

[65] Reilly, T. The menstrual cycle and human performance: an overview. *Biol. Rhythm Res.* **31** (1), 29–40 (2000). 10.1076/0929-1016(200002)31:1;1-0;FT088 11543399

[66] Tourville, T. W. et al. Evaluation of an algorithm to predict Menstrual-Cycle phase at the time of injury. *J. Athl Train.* **51** (1), 47–56. 10.4085/1062-6050-51.3.01 (2016). [published Online First: 25 January 2016]. 26807868 10.4085/1062-6050-51.3.01 PMC4851128

[67] Brukner, P. & Khan, K. *CLINICAL SPORTS MEDICINE* (McGraw-Hill Education, 2017).

[68] MacMillan, C. et al. The association between menstrual cycle Phase, menstrual Irregularities, contraceptive use and musculoskeletal injury among female athletes: A scoping review. *Sports Med.* **54** (10), 2515–2530. 10.1007/s40279-024-02074-5 (2024). [published Online First: 31 August 2024]. 39215933 10.1007/s40279-024-02074-5 PMC11467081

[69] Shultz, S. J. et al. Changes in serum collagen markers, IGF-I, and knee joint laxity across the menstrual cycle. *J. Orthop. Res.* **30** (9), 1405–1412. 10.1002/jor.22093 (2012). [published Online First: 2 March 2012]. 22389002 10.1002/jor.22093 PMC3371148

[70] Iwańska, D. et al. The effect of the menstrual cycle on collagen metabolism, growth hormones and strength in young physically active women. *Biol. Sport*. **38** (4), 721–728. 10.5114/biolsport.2021.107314 (2021). [published Online First: 30 June 2021]. 34937983 10.5114/biolsport.2021.107314 PMC8670799

[71] Spangenburg, E. E. et al. Regulation of physiological and metabolic function of muscle by female sex steroids. *Med. Sci. Sports Exerc.* **44** (9), 1653–1662 (2012). 22525764 10.1249/MSS.0b013e31825871fa PMC3422439

