# Supplementary figures and images for: The influence of the menstrual cycle on muscle injuries - a systematic review and meta-analysis
Source: Sci Rep. 2026 Jan 21;16:3035. doi: 10.1038/s41598-026-36763-0 (PMC12827947; doi:10.1038/s41598-026-36763-0)

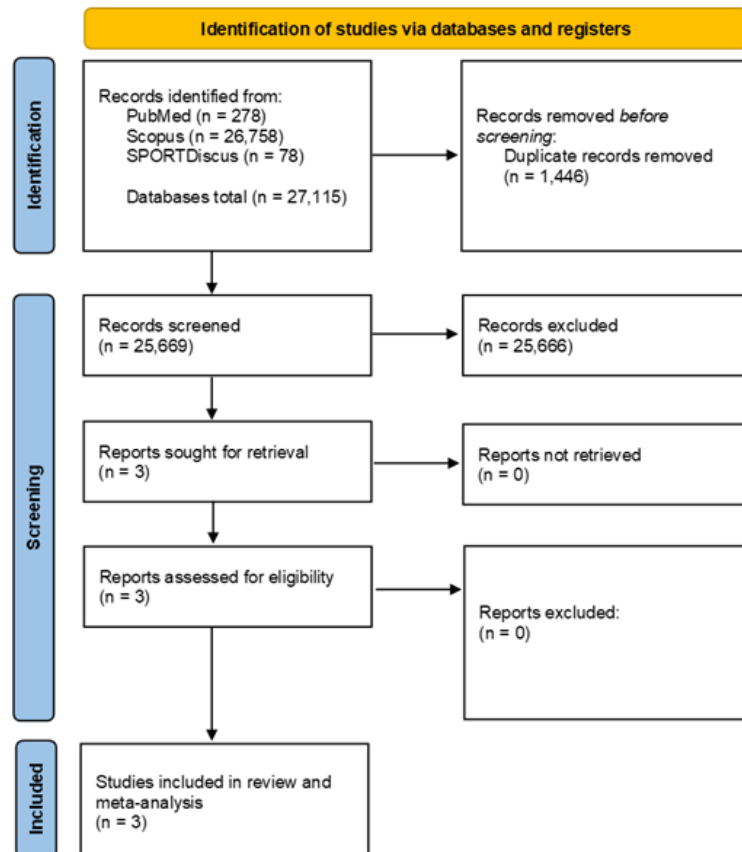

Supplement: Supplementary file 1 — Supplementary Material 1 [file 41598_2026_36763_MOESM1_ESM.pdf]
